# Prevalence of metabolic syndrome in Brazilian adults: a systematic review

**DOI:** 10.1186/1471-2458-13-1198

**Published:** 2013-12-18

**Authors:** Fernanda de Carvalho Vidigal, Josefina Bressan, Nancy Babio, Jordi Salas-Salvadó

**Affiliations:** 1 Postgraduate Program in Nutrition Science, Department of Nutrition and Health, Federal University of Viçosa, Viçosa, Brazil; 2 Human Nutrition Unit, Department of Biochemistry and Biotechnology, University Hospital Sant Joan de Reus, IISPV, Faculty of Medicine and Health Sciences, Rovira i Virgili University, Reus, Spain; 3 CIBERobn Physiopathology of Obesity and Nutrition, Institute of Health Carlos III, Madrid, Spain

**Keywords:** Metabolic syndrome, Prevalence, Brazil

## Abstract

**Background:**

The metabolic syndrome (MS) is a complex of risk factors for cardiovascular disease. This syndrome increases the risk of diabetes, cardiovascular disease and all-cause mortality. It has been demonstrated that the prevalence of MS is increasing worldwide. Despite the importance of MS in the context of metabolic and cardiovascular disease, few studies have described the prevalence of MS and its determinants in Latin America. The present study aims to assess studies describing the prevalence of MS in Brazil in order to determine the global prevalence of the syndrome and its components.

**Methods:**

Systematic review. Searches were carried out in PubMed and Scielo from the earliest available online indexing year through May 2013. There were no restrictions on language. The search terms used to describe MS were taken from the PubMed (MeSH) dictionary: “metabolic syndrome x”, “prevalence” and “Brazil”. Studies were included if they were cross-sectional, described the prevalence of MS and were conducted in apparently healthy subjects, from the general population, 19-64 years old (adult and middle aged) of both genders. The titles and abstracts of all the articles identified were screened for eligibility.

**Results:**

Ten cross-sectional studies were selected. The weighted mean for general prevalence of MS in Brazil was 29.6% (range: 14.9%-65.3%). Half of the studies used the criteria for clinical diagnosis of MS proposed by the National Cholesterol Education Program Adult Treatment Panel III (NCEP-ATP III) (2001). The highest prevalence of MS (65.3%) was found in a study conducted in an indigenous population, whereas the lowest prevalence of MS (14.9%) was reported in a rural area. The most frequent MS components were low HDL-cholesterol (59.3%) and hypertension (52.5%).

**Conclusions:**

Despite methodological differences among the studies selected, our findings suggested a high prevalence of MS in the Brazilian adult population.

## Background

The metabolic syndrome (MS) is a complex of interrelated risk factors for cardiovascular disease and diabetes. These factors include hyperglycemia, hypertension, high triacylglycerol levels, low HDL-cholesterol (HDL-c) levels, and abdominal obesity [1]. Separately the MS components increase the risk of diabetes, cardiovascular disease and all-cause mortality, but the full syndrome is associated with risk increases that are greater than the sum of the risk of each feature [2]. It has been reported that the association of MS with cardiovascular disease increases total mortality 1.5 times and cardiovascular death 2.5 times [3]. People with MS also have a 5-fold higher risk of developing type 2 diabetes [4].

The underlying cause of MS continues to challenge the experts. However, insulin resistance and abdominal obesity are postulated to be the key components. Genetic predisposition, physical inactivity, smoking, an unhealthy dietary pattern, ageing, proinflammatory state and hormonal changes may also have a causal effect. Their role, however, may depend on ethnic group [3-5]. The origin of all those metabolic disorders can be explained by a proinflammatory state derived from excessive caloric intake and overnutrition, and, perhaps, other chronic inflammatory conditions. This hypothesis asserts that this proinflammatory state, being characterized by an increase in inflammatory mediators, induce insulin resistance and leads to oxidative stress, with the potential to impair several biological pathways inducing insulin resistance. Therefore, insulin resistance could act as the common link among all the components of MS [6].

It has been demonstrated that the prevalence of MS is increasing worldwide, and for the adult population is estimated to be about 20 to 25%, largely the result of greater obesity and sedentary lifestyles [1,4]. In the United States of America, the prevalence of MS estimated in adults from the National Health and Nutrition Examination Survey (NHANES) decreased from 25.5% in 1999/2000 to 22.9% in 2009/2010 [7]. Studies conducted in Latin American populations from Chile, Colombia, Mexico, Peru and Venezuela revealed a high prevalence of MS which ranged from 12.3% to 42.7% depending on the criteria for clinical diagnosis and the characteristics of the study population [8-12].

Despite of the importance of MS in the context of metabolic and cardiovascular disease, in Brazil, few studies have described the prevalence of MS and its determinants, hence restricting the quality of information available on the magnitude of this problem in the country. Therefore, to help provide a clearer picture of the current situation in Brazil, we aimed to systematically review the available epidemiological data on the prevalence of MS in the Brazilian adult population.

## Methods

### Search strategy and eligibility criteria

An electronic bibliographic index (PubMed) and a multidisciplinary database for Ibero-America (Scielo) were searched from the earliest available online indexing year through May 2013, with no language restrictions. The search terms used to describe MS were taken from the PubMed (MeSH) dictionary: “metabolic syndrome x” [MeSH] AND “prevalence” [MeSH] AND “Brazil” [MeSH] refined by ages (adult: 19-44 years AND middle aged: 45-64) in “All Fields” as tag terms. The key words used in Scielo were the same as those used in PubMed (MeSH) without filter restrictions.

**Figure 1:**
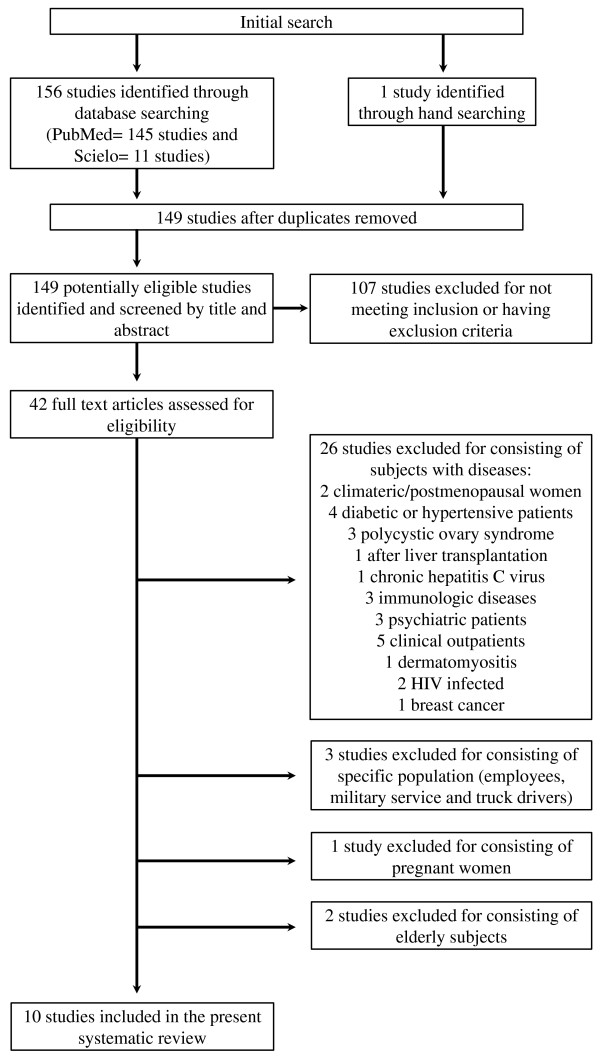
Flow chart of articles included in the present systematic review.

Studies were included if they were cross-sectional, described the prevalence of MS and were conducted in Brazilian populations. We restricted this review to studies on healthy adults and/or on the general population. Studies were excluded if they were conducted in subjects with diseases, in pregnant women, in the elderly (≥65 years) or in a specific population (Figure 1).

### Study selection and data extraction

The title and abstracts of all the articles identified were screened for eligibility. All potentially relevant titles and abstracts were selected for full text examination. To be included in the review, a study had to: 1) be cross-sectional; 2) focus on MS prevalence; 3) examine a healthy population; 4) examine a general adult population (19 to 64 years old) of both sexes; 5) provide sufficient information to accomplish the objectives of the present systematic review. Although the focus of our review was the adult population, some of the articles selected included both adult and elderly subjects, and therefore some subjects were more than 65 years old.

A total of 156 studies containing data on the prevalence of MS in Brazil were automatically identified by applying the aforementioned search terms and 1 study was selected by hand searching. The selection processes for the articles are shown in Figure 1.

Of the 145 studies identified in PubMed, 105 were excluded because they did not meet the inclusion criteria or had exclusion criteria. Of the 40 articles screened (titles and abstracts), 31 were excluded for the following reasons: 25 were conducted on subjects with diseases, 3 were carried out on specific populations, 1 comprised pregnant women and 2 consisted of elderly subjects. Therefore, 9 studies from this database were included in our systematic review.

Of the 11 studies identified by Scielo, 8 were excluded because they were duplicates, 2 did not meet the inclusion criteria or had exclusion criteria, and 1 was conducted on subjects with diseases. Therefore, no studies from this database were included.

One study was selected by hand searching, so a total of 10 studies were finally selected for the present systematic review.

Once the articles had been selected, the following significant data were extracted: authors and year of publication, study location (city, region), number of participants (W/M), age range, measurements, criteria for diagnosis of MS, overall prevalence of MS (%), overall prevalence of individual components of MS (%) and relevant findings/associations.

### Outcome

The weighted mean prevalence observed in the present systematic review was calculated as follows: sum of the number of cases in the studies considered ÷ sum of the number of subjects in all studies considered × 100 [13].

## Results of the studies reviewed

We summarize the results of ten cross-sectional studies selected for the present systematic review (Table 1). The studies had been published in scientific journals with impact factors ranging from 0.4 [14,15] to 5.509 [16].

**Table 1:** Characteristics of cross-sectional studies that evaluated the prevalence of metabolic syndrome in the Brazilian adult population

**Authors and publication year (Ref. no.)**	**City, region**	**Number of participants (W, M)**	**Age range (years)**	**Measurements**	**Criteria for diagnosis of MS**	**Overall prevalence of MS (%)**	**Overall prevalence of individual components of MS (%)**	Relevant findings/Associations*
Dutra et al., 2012 [18]	Brasília, Federal District	2130 (72.5% W, 27.5% M)	≥18	WC: at the midpoint between the iliac crest and the lowest rib	IDF and AHA/NHLBI harmonized criteria (2009)	32.0	AO: N/A	Logistic regression model adjusted for age:
				Fasting G, TAG and HDL-c BP: two measurements			High G: N/A	Age 25-34 years: female: PR = 2.06; 95% CI 1.13-3.74; male: PR = 3.35; 95% CI 1.27-8.88
							HT: N/A	Age 35-44 years: female: PR = 3.62; 95% CI 2.04-6.44; male: PR = 4.87; 95% CI 1.94-12.20
							High TAG: N/A	Age 45-54 years: female: PR = 5.42; 95% CI 3.08-9.55; male: PR = 7.60; 95% CI 3.08-18.70
							Low HDL-c: N/A	Age 55-64 years: female: PR = 6.57; 95% CI 3.74-11.55; male: PR = 8.95; 95% CI 3.64-22.00
								Age ≥65 years: female: PR = 7.45; 95% CI 4.23-13.11; male: PR = 7.89; 95% CI 3.14-19.80
								BMI 25-29 kg/m^2^: female: PR = 4.29; 95% CI 3.21-5.75; male: PR = 4.52; 95% CI 2.64-7.74
								BMI ≥30 kg/m^2^: female: PR =7.04; 95% CI 5.32-9.31; male: PR =9.99; 95% CI 5.92-16.90
								Schooling 9-11 years: female: PR = 0.78; 95% CI 0.64-0.96
								Schooling ≥12 years: female: PR = 0.51; 95% CI 0.37-0.70
de Oliveira et al., 2011 [21]	Jaguapiru Indigenous Village, Dourados, Mato Grosso do Sul	606 (55.8% W, 44.2% M)	18-69	WC: at the midpoint between the iliac crest and the lowest rib	IDF	35.7	AO: 60.9	N/A
				Fasting G, TAG and HDL-c			High G: 11.4	
				BP: two measurements			HT: 40.3	
							High TAG: N/A	
							Low HDL-c: N/A	
Gronner et al., 2011 [17]	Southeastern region	1116 (64.5% W, 35.5% M)	30-79	WC: at the midpoint between the iliac crest and the lowest rib	NCEP-ATPIII (2005) and IDF	NCEP-ATPIII (2005): 35.9	AO (NCEP-ATPIII 2005): 56.2	Logistic regression model:
				Fasting G, TAG and HDL-c		IDF: 43.2	AO (IDF): 72.6	Age 40-49 years†: OR = 2.15; 95% CI 1.47-3.16
				BP: three measurements			High G: 13.3	Age 50-59 years†: OR = 2.68; 95% CI 1.85-3.89
							HT: 59.2	Age 60-69 years†: OR = 4.64; 95% CI 3.06-7.04
							High TAG: 16.8	Age 70-79 years†: OR = 4.77; 95% CI 3.11-7.32
							Low HDL-c: 76.3	Skin color white‡: OR = 1.65; 95% CI 1.28-2.14
								Schooling fundamental§: OR = 2.51; 95% CI 1.58-4.00
								BMI ≥25 kg/m^2^: OR = 5.68; 95% CI 4.30-7.51
Pimenta et al., 2011 [14]	Virgem das Graças and Caju, Jequitinhonha Valley, Minas Gerais	534 (49.4% W, 50.6% M)	≥18	WC: at the midpoint between the iliac crest and the lowest rib	NCEP-ATPIII (2005)	14.9	AO: 11.6	Poisson regression model:
				Fasting G, TAG and HDL-c			High G: 10.6	Female: PR = 2.20; 95% CI 1.33-3.62
				BP: three measurements			HT: 59.7	Obesity (BMI ≥30 kg/m^2^): PR = 3.03; 95% CI 2.05-4.48
							High TAG: 15.2	CRP ≥4º : PR = 1.56; 95% CI 1.05-2.31
							Low HDL-c: 44.1	HOMA-IR ≥4º quartile: PR = 1.92; 95% CI 1.28-2.88
								Age ≥60 years†: PR = 7.06; CI 95% 2.62-19.04
								Alcohol consumption (0.1 to 20 g ethanol/day): PR = 0.26; CI 95% 0.09-0.73
da Rocha et al., 2011 [22]	Porto Alegre and Planalto/ Nonoai, Rio Grande do Sul	150 (55.3% W, 44.7% M)	40-104	WC: at the midpoint between the iliac crest and the lowest rib	NCEP-ATPIII (2001)	65.3	AO: 64.7	N/A
				Fasting G, TAG and HDL-c			High G: 38.0	
				BP: N/A			HT: 64.7	
							High TAG: 48.7	
							Low HDL-c: 67.3	
Silva et al., 2011 [15]	São Paulo	287 (74.6% W, 25.4% M)	20-64	WC: at the midpoint between the iliac crest and the lowest rib	IDF	36.6	AO: N/A	Short height adjusted for age and gender: OR = 1.25; 95% CI 1.12-1.26
				Fasting G, TAG and HDL-c			High G: N/A	
				BP: two measurements			HT: N/As	
							High TAG: N/A	
							Low HDL-c: N/A	
Marquezine et al., 2008 [16]	Vitória, Espírito Santo	1561 (54.5% W, 45.5% M)	25-64	WC: at the midpoint between the iliac crest and the lowest rib	NCEP-ATPIII (2001)	25.4	AO: 16.3	Logistic regression model adjusted for age in female:
				Fasting G, TAG and HDL-c			High G: 21.4	Low social class: OR = 1.64
				BP: three measurements			HT: 46.6	
							High TAG: 30.9	
							Low HDL-c: 54.3	
Salaroli et al., 2007 [5]	Vitória, Espírito Santo	1630 (54.4% W, 45.6% M)	25-64	WC: at the natural waist or the lower curvature between the lowest rib and the iliac crest	NCEP-ATPIII (2001)	29.8	AO: N/A	N/A
				Fasting G, TAG and HDL-c			High G: N/A	
				BP: two measurements			HT: N/A	
							High TAG: N/A	
							Low HDL-c: N/A	
Velásquez-Meléndez et al., 2007 [19]	Virgem das Graças, Jequitinhonha Valley, Minas Gerais	251 (53.4% W, 46.6% M)	18-88	WC: at the midpoint between the iliac crest and the lowest rib	NCEP-ATPIII (2001)	21.6	AO: 26.7	Logistic regression model adjusted for BMI, age and schooling:
				Fasting G, TAG and HDL-c			High G: 6.0	BMI ≥25 kg/m^2^: OR = 21.14; 95% CI 8.43-50.01
				BP: three measurements			HT: 62.5	Age 30-42 years†: OR = 3.15; 95% CI 1.08-9.18
							High TAG: 22.3	Age 43-59 years†: OR = 5.18; 95% CI 1.38-19.41
							Low HDL-c: 37.1	Age 60-88 years†: OR = 17.58; 95% CI 3.45-49.51
de Oliveira et al., 2006 [20]	Cavunge, Bahia	240 (57.5% W, 42.5% M)	25-87	WC: at the umbilical level	NCEP-ATPIII (2001)	30.0	AO: 31.7	Age ≥45 years: PR = 2.60; 95% CI 1.61-4.21
				Fasting G, TAG and HDL-c			High G: 15.8	Age ≥55 years: PR = 3.00; 95% CI 1.84-4.89
				BP: two measurements			HT: 57.1	
							High TAG: 19.6	
							Low HDL-c: 70.4	

Abbreviations: AHA/NHLBI, American Heart Association/National Heart, Lung, and Blood Institute; AO, abdominal obesity; BP, blood pressure; BMI, body mass index; CRP, C-reactive protein; G, glucose; HDL-c, HDL-cholesterol; high G, hyperglycemia; high TAG, hypertriglyceridemia; HOMA-IR, Homeostasis Model Assessment of Insulin Resistance; HT, hypertension; IDF, International Diabetes Federation; M, men; MS, metabolic syndrome; N/A, information not available; NCEP-ATP III, National Cholesterol Education Program Adult Treatment Panel III; PR, prevalence ratio; TAG, triacylglycerol; W, women; WC, waist circumference.

*Only statistically significant associations are shown (P < 0.05).

†Reference group for age <30 years.

‡Reference group for non-white skin color.

§Reference group for higher educational level.

The studies were conducted in several populations, and various criteria had been used to diagnose MS. Of the ten cross-sectional studies selected, five had been carried out in urban populations [5,15-18], 3 in rural populations [14,19,20] and 2 in indigenous populations [21,22]. According to demographic census by 2010 [23], in Brazil the indigenous population was 817,963 (0.43%) for a total population of 190,755,799. Therefore, it was important to include the indigenous population in the present review since they are part of the Brazilian population. Five of the ten studies used the criteria for diagnosing MS proposed by the National Cholesterol Education Program Adult Treatment Panel III (NCEP-ATP III) (2001) [5,16,19,20,22]; two the criteria of the International Diabetes Federation (IDF) [15,21]; one the criteria of the NCEP-ATP III (2005) [14]; one the harmonized criteria of the IDF and American Heart Association/National Heart, Lung, and Blood Institute (AHA/NHLBI) [18]; and one study used both IDF and NCEP-ATP III (2005) criteria [17]. Five studies [5,14,16,19,20] used a mercury sphygmomanometer; de Oliveira et al. [21] used an aneroid sphygmomanometer; whereas Dutra et al. [18] and Gronner et al. [17] used an automatic instrument (OMRON®) for measuring blood pressure. The two remaining studies [15,22] did not mention how blood pressure was measured. Nine studies reported the number of measurements carried out (two [5,15,18,20,21] or three [14,16,17,19]). Seven studies mentioned the measurement site of blood pressure (right arm [14,19,21,22], left arm [16,20] or nondominant arm [5]). Of the ten studies, eight measured the waist circumference at the midpoint between the iliac crest and the lowest rib [14-19,21,22]; one at the lower curvature between the lowest rib and the iliac crest [5]; and one at the umbilical level [20] (Table 1).

### Prevalence of metabolic syndrome in Brazil

The studies selected [5,14-22] in this systematic review comprised 8,505 subjects, 60.8% of whom were women and 39.2% men. Nine studies had more women than men [5,15-22], whereas one had similar numbers (50.6% men and 49.4% women) [14].

In nine studies, the general prevalence of MS (unadjusted for age and/or gender) was reported [5,14-16,18-22]. In two of these the prevalence was adjusted for age [19,20], and in one it was adjusted for age and gender [15]. One study did not report the general prevalence and only described the prevalence adjusted for age and gender [17]. In the studies that mentioned rates of prevalence, the weighted mean for general prevalence of MS was 29.6% [5,14-16,18-22]. In the studies that mentioned rates of prevalence using the NCEP-ATP III (2001) criterion, the weighted mean for general prevalence of MS was 28.9% [5,16,19,20,22]. The weighted mean for age adjusted prevalence was 22% [19,20] and the weighted mean for age and gender adjusted prevalence was 41.3% [15,17], using the NCEP-ATP III (2001) and the IDF criteria, respectively. The weighted mean for general prevalence of MS according to region was 29.8%, 20.1% and 41.5% in urban [5,15,16,18], rural [14,19,20], and indigenous [21,22] populations, respectively. Taking into account that the indigenous population represents only 0.43% of the total Brazilian population, we calculated the weighted mean for general prevalence of MS excluding the studies [21,22] conducted in indigenous population. The weighted prevalence of MS without taking into account the indigenous population was 28.3%, only 1.3% lower than those calculated using the indigenous population studies (29.6%).

MS was most prevalent (65.3%) in a study conducted in the indigenous population of Rio Grande do Sul, using the NCEP-ATP III (2001) criterion for diagnosing MS [22]. The lowest prevalence of MS (14.9%) was reported in Virgem das Graças and Caju, a rural area in the Jequitinhonha Valley (Minas Gerais), using the NCEP-ATP III (2005) criterion [14].

Nine studies reported the prevalence of MS by gender [5,14,16-22]. In five studies [14,19-22] the prevalence of MS was higher in women than in men. In four other studies [5,16-18] no difference was observed in the prevalence of MS between genders. The difference in prevalence between genders ranged between 0.2% [17] and 44.7% [22].

Nine studies reported the prevalence of MS by age [5,14,16-22]. In seven of these [5,14,16-20], the prevalence of MS increased with age, whereas in the other two [21,22] this was not demonstrated. The prevalence of MS was highest verified in subjects more than 50 years old. In contrast, prevalence was generally lowest in those under 30, with the exception of the indigenous population of Mato Grosso do Sul where the lowest prevalence (9.6%) was observed in subjects between 60 and 69 [21].

### Prevalence of the metabolic syndrome components

The prevalence of the individual components of MS among the Brazilian population varied considerably between studies (Table 1). In six studies [14,16,17,19,20,22], the overall prevalence of individual components of MS was reported, whereas de Oliveira et al. [21] reported only the prevalence of abdominal obesity, hyperglycemia and hypertension. The overall weighted mean prevalence (range) by component was as follows: abdominal obesity 38.9% (11.6%[14]-72.6%[17]); hyperglycemia 16% (6%[19]-38%[22]); hypertension 52.5% (40.3%[21]-64.7%[22]); hypertriglyceridemia 24% (15.2%[14]-48.7%[22]); low HDL-c 59.3% (37.1%[19]-76.3%[17]).

The prevalence of individual components of MS by gender was reported in three studies [14,19,21]. In addition, three other studies described the prevalence of MS components by age and gender group [16,17,20]. In general, the prevalence of abdominal obesity and low HDL-c was higher in females, whereas prevalence of hypertension was higher in males. Generally, the prevalence of the individual components was higher in subjects more than 45 years old, except for the low HDL-c component.

#### Abdominal obesity

Seven studies reported the prevalence of abdominal obesity [14,16,17,19-22]. In these seven studies, the weighted mean prevalence of abdominal obesity was 38.9%. The highest prevalence of abdominal obesity (72.6%) was found in the urban population of São Paulo using the IDF criterion [17], whereas the lowest prevalence (11.6%) was observed in the rural population of Minas Gerais using the NCEP-ATP III (2005) criterion [14].

#### Hyperglycemia

The prevalence of hyperglycemia was mentioned in seven studies [14,16,17,19-22]. The weighted mean prevalence of hyperglycemia was 16%. The prevalence (38%) was highest in the indigenous population of Rio Grande do Sul [22], and lowest (6%) in the rural population of Minas Gerais [19]. Both studies used the NCEP-ATP III (2001) criteria.

#### Hypertension

The prevalence of hypertension was reported in seven studies [14,16,17,19-22]. The weighted mean prevalence of hypertension was 52.5%. Hypertension was shown to be the most prevalent (64.7%) in the indigenous population of Rio Grande do Sul [22]. The prevalence (40.3%) was lowest in the indigenous population of Mato Grosso do Sul [21].

#### Hypertriglyceridemia

The weighted mean prevalence of hypertriglyceridemia was 24% in six studies [14,16,17,19,20,22]. Hypertriglyceridemia was most prevalent (48.7%) among the indigenous population of Rio Grande do Sul [22], and least prevalent (15.2%) in the rural population of Minas Gerais [14].

#### Low HDL-cholesterol

Six studies reported the prevalence of the low HDL-c component of the MS [14,16,17,19,20,22]. The weighted mean prevalence of low HDL-c was 59.3%. The highest prevalence of this component was 76.3% in the urban population of São Paulo [17], and the lowest (37.1%) was found in the rural population of Minas Gerais [19].

### Factors associated with metabolic syndrome

The risk of MS increased significantly with age, body mass index (BMI) and such conditions as having a white skin color (compared with a non-white skin color), being female, belonging to a low social class, and belonging to the highest quartiles of C-reactive protein and Homeostasis Model Assessment of Insulin Resistance levels (Table 1). The strongest association was found with BMI ≥25 kg/m^2^ (OR = 21.14; 95% CI 8.43-50.01) [19], followed by age with subjects aged between 60 and 88 years old compared with those under 30 (OR = 17.58; 95% CI 3.45-49.51) [19].

Two studies reported factors that protected against the risk of MS. The education level in females [18], and moderate alcohol consumption (0.1 to 20 g ethanol/day) in males and females [14] were inversely related to MS prevalence.

## Discussion

The present systematic review provides data on the prevalence of MS in the Brazilian adult population. According to this review, the weighted mean for the general prevalence of MS was between 28.9 and 29.6% according to the criteria used to define MS. This observed prevalence was slightly higher than the prevalence estimated around the world (between 20% and 25%) [4]. In fact, the mean prevalence of MS in Brazil was higher than that reported in such European countries as Portugal (27.6%) [24], Spain (26.6%) [25], France (25% in males and 15.3% in females) [26] and Italy (28% in males and 26% in females) [27]. It was also higher than in the United States of America (22.9%) [7] and some Latin American countries: Mexico (26.6%) [8] and Peru (18.1%) [10]. This mean prevalence of MS was higher than that observed in the CARMELA study, conducted in seven Latin American countries, and with a prevalence ranging from 14% to 27% [28]. Prevalences similar to the one we reported in Brazil were found in Chile (29.5%) [12] and North Africa (30%) [29]. The mean prevalence we found in Brazil, however, was lower than that reported in Asia-China (33.9%) [30], Turkey (36.6%) [31], and Iran (30.1% in males and 55% in females) [32]-and some other Latin American countries: Colombia (34.8%) [11] and Venezuela (35.3%) [9].

The highest prevalence (65.3%) of MS in Brazil was reported in the indigenous population [22]. Likewise, when analyzed by region (urban, rural and indigenous), the highest weighted mean for general prevalence of MS was found in the indigenous population (41.5%) [21,22], which suggests that these individuals are at higher risk of cardiovascular disease and diabetes. Kuang-Yao Pan et al. [33] demonstrated that indigenous groups living in the northern Ecuadorian Amazon had a 30% higher probability of mortality and a 62% higher incidence rate of all-cause morbidity than colonists. According to the First National Survey of Indigenous People’s Health and Nutrition, non-pregnant women presented the following risk factors: overweight (30.3%), obesity (15.8%) and hypertension (13.2%) [34].

It is evident that substantial socioeconomic and demographic changes have occurred in the Brazilian population over the past decades and the transition from a rural to an urban lifestyle has been associated with a deterioration in the metabolic profile because of adverse changes in lifestyle habits [19]. Observed disparities in health indicators underscore that basic healthcare and sanitation services are not yet as widely available in Brazil’s indigenous communities as they are in the rest of the country [34].

In the present review, the most frequent MS component was low HDL-c (59.3%). The same results were found in Venezuela (65.3%) [9], Colombia (96.1%) [11] and Peru (females = 60.9%) [10]. In other studies, the most frequent MS component was abdominal obesity: United States of America (56.1%) and Chile (41.1%) [12]. In contrast, the least frequent MS component in Brazil was hyperglycemia (16%). This was also the rarest MS component observed in studies conducted in Colombia (3.9%) [11], the United States of America (19.9%) [7] and Peru (5% in females and 5.4% in males) [10]. In the Chinese population, the least frequent MS component was low HDL-c (5% in females and 12.9% in males) [30].

The general prevalence of MS in Brazil, according to this review, ranged from 14.9% [14] to 65.3% [22], the differences in MS prevalence being as high as 50.4%. The differences were also notable in terms of the prevalence of MS components with the abdominal obesity component showing the highest difference (61%). The study of MS has been hampered by the lack of consensus on its definition, the cutoff points used for its respective components, and how waist circumference and blood pressure should be determined. This has an impact on clinical practice and health policy [3]. Barbosa et al. [35] conducted a cross-sectional study in a population subgroup of 1439 adults in the city of Salvador in Brazil, and showed that the cutoff points for waist circumference proposed by NCEP-ATP III [36] were inappropriate and underestimated the prevalence of MS in the population studied, particularly in men. Different cutoff points for the waist circumference and the measurement site of waist circumference make it difficult to compare studies. Our results showed that in most studies [14-19,21,22] waist circumference was measured at the midpoint between the iliac crest and the lowest rib. However, Salaroli et al. [5] took measurements at the natural waist or the lower curvature between the lowest rib and the iliac crest, and de Oliveira et al. [20] at the umbilical level. Moreover, different techniques and instruments were also used to measure blood pressure. Another factor that limits the comparability between studies is the different source and type of population studied. For instance, five of the studies selected were conducted in urban populations [5,15-18], three in rural populations [14,19,20] and two in indigenous populations [21,22]. Nevertheless, we selected only studies that evaluated apparently healthy adults from the general population in order to make it possible to compare studies, despite the variability in the populations studied. Nine studies had more women than men [5,15-22], whereas one had similar numbers of men and women [14].

In order to diagnose MS, half of studies [5,16,19,20,22] used the NCEP-ATP III (2001) criterion. There is no consensus about the best criterion for diagnosing MS in clinical practice. Recently, IDF and AHA/NHLBI representatives have held discussions in an attempt to resolve the remaining differences between MS definitions and they have recognised that the risk associated with a particular waist circumference measurement will depend on the population. The harmonized criterion proposed by IDF and AHA/NHLBI will enable countries to be compared in the future and should be used as a unifying worldwide consensus definition for MS [1]. Apart from the methodological differences between studies, the variability in the prevalence of MS observed in our review between populations could be explained by demographic, epidemiological and nutritional transitions, as well as environmental and social influences, and ethnic differences [18].

Like other reviews, this study has some limitations, so its findings should be interpreted with caution. The multicultural characteristics and the demographic and epidemiological variability in the Brazilian population make it difficult to generalize the findings of this study in Brazil. Further limitations are the different criteria used to diagnose MS, the different measurement sites and cutoff points for waist circumference and the cutoff points for hyperglycemia. Hence, the need for more specific and structured research on the prevalence of MS and its determinants in Brazil are imperative.

## Conclusions

To our knowledge, this is the first published systematic review assessing studies on the prevalence of MS and its determinants in the Brazilian adult population. Despite the methodological differences and the lack of consensus on criteria for MS diagnosis, our systematic review indicates a high prevalence of MS in the healthy Brazilian adult population. Criteria for MS diagnosis need to be standardized and suitable cutoff points for individual MS components in Brazil defined if the precise scope of this public health problem in Brazil is to be determined. Information about how the clinical MS components are distributed and how they are related could provide greater insight into MS and contribute to the planning and implementation of public health strategies. Therefore, primary preventive care could be used to reduce its prevalence and impact on human health.

## Abbreviations

AHA/NHLBI: American Heart Association/National Heart, Lung, and Blood Institute; BMI: Body mass index; HDL-c: HDL-cholesterol; IDF: International Diabetes Federation; MS: Metabolic syndrome; NCEP-ATP III: National Cholesterol Education Program Adult Treatment Panel III; NHANES: National Health and Nutrition Examination Survey.

## Competing interests

The authors declare that they have no competing interests.

## Authors’ contributions

JB and JSS directed the present study. All the authors contributed to the study concept and design. FCV helped with article searches, review and selection. All the authors contributed to the analysis and interpretation of data and drafting of the manuscript. JSS and NB worked as methodological advisors. All authors read and approved the final manuscript.

## Pre-publication history

The pre-publication history for this paper can be accessed here:

http://www.biomedcentral.com/1471-2458/13/1198/prepub
